# Regional variations of perceived problems in ambulatory care from the perspective of general practitioners and their patients - an exploratory focus group study in urban and rural regions of northern Germany

**DOI:** 10.1186/s12875-017-0637-x

**Published:** 2017-05-25

**Authors:** H. Hansen, N. J. Pohontsch, L. Bole, I. Schäfer, M. Scherer

**Affiliations:** Department of General Practice/Primary Care, University Medical Center Hamburg-Eppendorf, Hamburg, Germany

**Keywords:** Problems in ambulatory care, Primary care, Focus groups, Qualitative research, Regional variations, General practitioner, Patient

## Abstract

**Background:**

Patients from rural and urban regions should have equitable access to health care. In Germany, the physician-patient-ratio and the supply of medical services vary greatly between urban and rural areas. The aim of our study was to explore the regional variations of the perceived health care problems in ambulatory care from the perspective of affected professionals and laypersons i.e. general practitioners and their patients.

**Methods:**

We conducted 27 focus groups with general practitioners (*n* = 65) and patients (*n* = 145) from urban areas, environs and rural areas in northern Germany. Discussions were facilitated by two researchers using a semi-structured guideline. The transcripts were content analyzed using deductive and inductive categories.

**Results:**

General practitioners and patients reported problems due to demographic change and patient behaviour, through structural inequalities and the ambulatory reimbursement system as well as with specialist care and inpatient care. A high physician density, associated with high competition between general practitioners, a high fluctuation of patients and a low status of general practitioners were the main problems reported in urban areas. In contrast, participants from rural areas reported an insufficient physician density, a lack of young recruits in primary care and a resulting increased workload as problematic. All regions are concerned with subjectively inadequate general practitioners’ budgets, insufficiently compensated consultations and problems in the cooperation with specialists and inpatient care institutions. Most problems were mentioned by GPs and patients alike, but some (e.g. high competition rates in urban regions and problems with inpatient care) were only mentioned by GPs.

**Conclusions:**

While many problems arise in urban regions as well as in rural regions, our results support the notion that there is an urgent need for action in rural areas. Possible measures include the support of telemedicine, delegation of medical services and reoccupation of vacant practices. The attractiveness of working in rural areas for general practitioners, specialists and clinicians must be increased by consolidating and expanding rural infrastructure (e.g. child care and cultural life). The above mentioned results also indicate that the ambulatory reimbursement system should be examined regarding the reported inequalities. Measures to further enhance the cooperation between general practitioners, specialists and inpatient care should be taken to solve supra-regionally reported problems. Problems showing regional variations indicate the need for measures to balance these variations between the regions.

This is the first German study to analyze subjective views of the stakeholders concerned on regionally variating problems in ambulatory care. Further studies are needed to quantify the extent of the identified problems and differences. A corresponding survey is currently under way.

## Background

One of the German government’s major aims is the provision of equitable access to medical services and health care for all citizens. Continuous, comprehensive, demand-oriented and close to home health care is one of the most important achievements of the German health care system [1]. Primary care delivered by a general practitioner (GP) usually constitutes the first contact between a patient and the health care system in Germany. Despite the fact that access to health care is generally provided for almost all citizens, access to primary health care is not equally easy for everyone. The physician-patient-ratio and the supply of certain medical services vary greatly between urban and rural areas, with rural areas experiencing a growing shortage of medical personnel and services [2, 3]. General practices are particularly unevenly distributed, with urban areas having a higher density of GPs and shorter distances than rural areas and especially sparsely populated districts in eastern Germany having the fewest GPs [2–6]. The density of GPs, specialists and psychotherapists is an important determinant for their utilization [7], and accessibility (e.g. traveling time) may determine the choice of the provider [8]. Thus, the uneven distribution of medical personnel and services is thought-provoking. These distribution phenomena do not exist in Germany exclusively but also poses a growing problem in e.g. England [9], the U.S. [10–12], Japan [13] and Australia [14].

Accessibility and availability problems or variations are not only found in the general practice setting, but also apply to the services of specialists and hospitals [2, 15]. A study by Koller et al. showed that persons living in urban areas had a higher chance of consulting a specialist [16]. Thode et al. reported that patients from rural areas visit their GPs as often as patients from urban areas, however, they have a lower number of contacts to different specialists in Germany [17]. Furthermore, residence in a rural area increases the frequency of ambulatory care sensitive hospitalizations slightly [18]. The (re-)admittance-to-hospital rate is higher and duration of the stay longer for palliative care patients living in rural areas than those living in urban areas of southern Germany [19].

GPs are also affected by regional differences in health care supply. There are differences in rural and urban GPs’ clientele and workload in Germany. The proportion of older and multimorbid patients is higher in rural areas than in urban areas [20, 21]. Average GPs from urban areas work 4 h less per week than their rural colleagues who see approx. 38 patients more per week [22]. Rural GPs perform more medical procedures than their urban counterparts, including procedures which might indicate their role in covering for missing specialists [23]. Rural patients receive lifestyle counseling for cardiovascular disease prevention through their GPs less often, due to a lack of time [24]. The higher workload of GPs from rural areas is amplified by the lack of young general practitioners. Being a GP in rural areas is associated with many prejudices e.g. low income, 24-hour accessibility and overtime or social control by the rural population [25–27]. When questioning medical students in Germany about their occupational perspectives and expectations the students consider urban areas as attractive places to work, whereas economically underdeveloped rural areas are highly unattractive [28].

The above mentioned points underline that provision of (primary) care is in some aspects different in rural and urban areas. Although this is usually considered as problematic, previous studies do not offer a comprehensive reflection of the affected persons’ (patients and GPs) experiences in ambulatory care. To our knowledge there is a paucity of research that enquires about regional variations in problems in ambulatory care from GPs’ and patients’ points of view. Additional problems might exist which are not yet identified by previous quantitative and qualitative studies especially from the patients’ point of view. It is unknown whether and how the abovementioned inequalities reflect in German patients’ and GPs’ experiences and perceptions of (problems in) ambulatory care. Do the presumably affected persons perceive problems in ambulatory care? Do they notice regional variations in ambulatory care and if so, do they feel negatively affected by these differences? Exploration of this experiences and perceptions is likely to better identify the most pressing subjective problems for those consuming and providing ambulatory care in rural and urban regions. Therefore, in the present study, the following research questions were addressed:

- Which ambulatory care problems currently exist from the perspective of general practitioners and their patients in northern Germany?
- How do these problems vary between urban, environ and rural regions?

## Methods

### Study design

This study is part of the mixed-methods project “Regional variation in primary medical care of Northern Germany (AVFN-Regional)”, which consists of a qualitative and a quantitative part: 1) an exploratory qualitative study (focus groups with GPs and patients) and 2) a survey to quantify regional differences in primary health care in northern Germany [29], ClinicalTrials.gov NCT02558322). This paper presents the results from the focus groups.

### Recruitment

The participants of the qualitative study were selected using criteria-controlled sampling [30]. Districts and cities in northern Germany were distributed to the three region types: “urban area”, “environs” and “rural area”, according to a map from the German Federal Institute for Research on Building, Urban Affairs and Spatial Development [31] (for further definition of region types see below). GPs and their patients were assigned to one of the regions depending on the GP’s area of residence.

The organization of the German health insurance system is characterized by the dual system of statutory health insurance and private health insurance. The majority of the German general practitioners are so-called statutory health insurance physician meaning that they are allowed to treat patients insured by the statutory and private health insurances and get reimbursed by statutory and private health insurances. As health care services provision differs between these two systems, we choose to focus on the paramount system of statutory health insurance (physicians). Therefore, GPs had to participate in the statutory health insurance system to be eligible for this study.

We randomly choose 1910 GPs (practicing in selected districts of Hamburg, Schleswig-Holstein, Lower Saxony and Mecklenburg-West Pomerania) from the database of the Association of Statutory Health Insurance Physicians to be invited to our study. GPs received a written invitation from our study center, while patients were recruited by their respective GPs. On an average day (no special or emergency consultation hours) the GPs consecutively asked all patients (18 years or older) to join until four patients (two patients younger than 50 years and two 50 years or older) gave their written consent to be contacted and included in the study by the study personnel. Exclusion criteria for patients were: not being a regular patient of the participating practices, not being able to participate in the focus groups, not being able to speak and/or read German, nursing home residency, lethal illnesses, and/or an insufficient ability to consent. All participants received financial compensation for their participation including a reimbursement of travel costs.

#### Definition of recruiting areas/region types

The German Federal Institute for Research on Building, Urban Affairs and Spatial Development differentiates between four types of settlement structures [31]: 1. District-free cities (urban municipalities) with at least 100.000 inhabitants, 2. urban districts [districts with at least 50% of the inhabitants living in cities with more than 20.000 inhabitants and at least 150 inhabitants/km^2^ plus districts with more than 150 inhabitants/km^2^without cities with more than 20.000 inhabitants], 3. rural districts with signs of agglomeration [districts with at least 50% of the inhabitants living in cities with more than 20.000 inhabitants but less than 150 inhabitants/km^2^ plus districts with less than 50% of the inhabitants living in cities with more than 20.000 inhabitants but less than 150 inhabitants/km^2^ without cities with more than 20.000 inhabitants] and 4. sparsely populated rural districts [districts with less than 50% of the inhabitants living in cities with more than 20.000 inhabitants and less than 100 inhabitants/km^2^). For the sake of simplicity in our study areas falling under the definition of district free cities were called ‘urban areas’, areas falling under the definition of urban districts (2.) and rural districts with signs of agglomeration (3.) were summed up under the term ‘environs’ and areas falling under the definition of sparsely populated rural districts (4.) are ‘rural areas’. We excluded all GPs from six cities with populations over 20.000 in rural areas in order to avoid a bias by ‘city doctors’ (assuming them to be working in an infrastructural environment more similar to environs and urban regions) in rural area focus groups.

### Sample

We conducted nine focus groups with 65 general practitioners (three focus groups in each area: urban areas (*n* = 24), environs (*n* = 19) and rural areas (*n* = 22)). 44 GPs were male and 21 female. Further descriptions of the participating GPs can be found in Table 1.

**Table 1:** Description of study participants: GPs (*n* = 65)

	Urban areas	Environs	Rural areas
Age in years:			
mean ± sd:	54,3 ± 7,7	50,6 ± 8,8	55,0 ± 9,7
Gender:			
Male	18	14	12
Female	6	5	10
Number of patients treated in practice in each quarter:			
Up to 749 patients	42%	5%	9%
750 patients and more	58%	95%	91%
Number of physicians working in practice:			
1	21%	48%	41%
2	46%	21%	46%
3	17%	21%	14%
4	13%	5%	-
5	4%	5%	-
Years of practice experience:			
mean ± sd	17,4 ± 10,0	12,4 ± 9,4	15,4 ± 9,2
Type of practice:			
Single practice	25,0%	52,6%	50,0%
Group practice (common accounting)	54,2%	42,1%	36,4%
Community practice (separate accounting)	20,8%	5,3%	13,6%

We also conducted nine focus groups with 65 younger patients [<50 years, three groups in each area: urban area (*n* = 23), environs (*n* = 20) and rural area (*n* = 22)] and nine focus groups with 80 older patients [≥50 years, three groups in each area: urban area (*n* = 26), environs (*n* = 25) and rural area (*n* = 29)]. Overall 69 patients were male and 76 female. Further descriptions of the participants can be found in Table 2.

**Table 2:** Description of study participants: patients (*n* = 145)

	Urban areas		Environs		Rural areas	
	18–49 years	50 years and older	18–49 years	50 years and older	18–49 years	50 years and older
Age:						
mean (sd)	34,9 ± 10,3	60,7 ± 8,2	37,9 ± 7,6	65,5 ± 8,0	40,1 ± 8,7	64,8 ± 9,1
Gender:						
Male	7	13	10	15	7	17
Female	16	13	10	10	15	12
Marital status:						
Married	17,4%	61,5%	45,0%	84,0%	50,0%	82,8%
Estranged (living in separate homes)	8,7%	-	5,0%	-	-	6,9%
Never married	60,9%	3,8%	45,0%	4,0%	22,7%	3,4%
Divorced	13,0%	23,1%	5,0%	4,0%	27,3%	3,4%
Widowed	-	11,5%	-	8,0%	-	3,4%
Education (in CASMIN grade):						
Grade 1 (low)	17,4%	42,3%	15,0%	40,0%	27,3%	34,5%
Grade 2 (medium)	65,2%	42,3%	75,0%	32,0%	54,5%	58,6%
Grade 3 (high)	17,4%	15,4%	10,0%	28,0%	18,2%	6,9%
Employment:						
Employed	87,0%	38,5%	95,0%	32,0%	63,6%	27,6%
Not employed	8,7%	23,1%	-	8,0%	18,2%	17,2%
Retired	18,2%	38,5%	5,0%	56,0%	18,2%	51,7%
Not reported	-	-	-	4,0%	-	3,4%
Chronic disease (yes):	47,8%	65,4%	50,0%	56,0%	50,0%	48,3%
Degree of disability (20–100, yes)^a^:	17,4%	42,3%	30,0%	24,0%	27,3%	27,6%

^a^‘Are you at the moment officially recognized as disabled (degree 20–100)?’: Yes/No

### Data collection

All focus groups were facilitated by at least two experienced moderators (post doc researchers; HH/NJP/IS). One focus group was moderated by IS and AS (M. Sc. Health Sciences), another two focus groups were moderated by HH or IS and AK (B. Sc. Health Sciences). The focus groups were conducted using an interview guide and lasted approx. 120 min. They were digitally recorded and logged by a student assistant. Different interview guidelines were used for focus groups with GPs and patients. The guidelines can be found in Table 3 (patients) and Table 4 (GPs).

**Table 3:** Guideline patient groups

Guideline patient groups	
Introduction: We invited you today to discuss *“*regional variations in primary care”.	
First of all, please tell us the reasons for your last three consultations with your GP.	
What did you expect your GP to do for you?	
What did your GP do?	
Which problems exist in primary care in your area of living?

**Table 4:** Guideline GP groups

Introduction: By conducting this study we would like to identify differences concerning the work of GPs in big cities, environs and rural areas with regard to typical expectations, needs and treatment requirements.	
Please describe the most common reasons for consultations in your practice.	
Which kinds of patients consult you most often?	
What do you think, what are the differences between working in your region of registration and bigger cities respectively rural areas? Please think of your own experiences of being a general practitioner or accounts of colleagues working for example in bigger cities or rural areas. Can you give some examples?	

### Transcription and protection of data privacy

The focus groups were transcribed verbatim, following designated transcription rules, by trained research assistants and checked for accuracy by HH. In order to protect the participants’ identities, all names were replaced by numbers and all potentially identifying details were changed.

### Data analysis

The transcripts were analyzed using the structuring qualitative content analysis according to Mayring [32]. Deductive categories were inferred from the interview guide. However, due to the exploratory character of the study, the main focus was placed on inductive categories derived from the material. HH, NJP, IS and LB analyzed the transcripts. All categories, category descriptions and examples were discussed and consented on by HH, NJP, IS and LB. The revised categories were discussed and consented with MS. After finalizing the category system, LB conducted a second round of coding to make sure that no relevant information was missed. To secure intersubjective comprehensibility and credibility of the analysis [33], the results were presented and discussed in a meeting of an interdisciplinary work group for qualitative methods (led by NJP). Data was managed using MAXQDA 11 (Verbi GmbH).

## Results

We identified six main categories of problems in ambulatory care from the perspective of GPs and their patients. The main categories and subcategories are summarized in Table 5. It shows which stakeholder (GP and/or patient) reported which problem in which region.

**Table 5:** Summary of main categories and subcategories of perceived problems in ambulatory care

No	Main category	Rural areas		Environs		Urban areas	
	Subcategory	GP	Pat	GP	Pat	GP	Pat
1	Problems due to demographic change						
	Aging patients and GPs	X		X	X	X	X
	Lack of young recruits in primary care	X	X	X	X		
2	Problems due to patient behaviour						
	Certain patient types are very time consuming	X		X	X	X	X
	Patients misjudge the necessity of treatment and consult their GPs too late	X	X	X	X		
	Patients misjudge the necessity treatment and consult their GPs about banalities					X	X
	Urban patients have less confidence in their GP’s abilities				X	X	X
3	Problems through structural inequalities						
	Not enough GPs in rural areas lead to long waiting times and crowded practices	X	X	X	X		
	The work-life balance of the GP is threatened by long working hours	X	X	X	X		
	(Too) many GPs in urban areas lead to high competition rates and to the poaching of patients					X	
	(Too) many GPs in urban areas lead to a higher fluctuation of patients					X	X
	A lack of parking spots leads to long commutes in urban areas (for GPs and patients)					X	X
	Long distances lead to long commutes in rural areas (for GPs and patients)	X	X	X	X		
4	Problems through the ambulatory compensation system						
	GPs’ budgets are not adequate	X	X		X	X	X
	Some consultations are not financially compensated for GPs	X		X		X	
5	Problems with specialist care						
	A lack of specialists leads to long waits for appointments	X	X	X	X	X	X
	GPs have to assume duties of the specialists	X	X	X	X		
	Specialists do not inform or inadequately inform their patients’ GPs			X		X	
6	Problems with inpatient care						
	GPs must provide follow-up care for patients discharged with still healing wounds	X		X		X	
	Staff shortage in hospitals in rural areas	X	X				
	Lack of cooperation/communication between GPs and hospitals	X		X		X	
	Discharge reports are incorrect or missing	X		X		X	
	Prescribed medications from hospitals must be reduced by the GP on an outpatient basis			X		X	
	GPs and patients consider some hospital therapies/diagnostics unnecessary	X		X			X

X = problems mentioned by interviewees in the respective area

### Category 1: Problems due to demographic change

An aging society was mentioned as a problem in all regions. The increasing average age of the patients causes a higher workload for the GPs. Aging patients often have multiple diseases, need much care and many home visits by their doctor. GPs have to compensate for a lack of social/familial support, especially in rural areas because of rural depopulation. The equally aging GP population is the other side of this problem. Patients and GPs fear a lack of GPs in the future. GPs mentioned that they could not compensate for the increasing numbers of patients, if there were even fewer GPs.

*“We are three people in [town in rural Schleswig-Holstein], one is about 70 […], I plan on working for a maximum of five more years and then there is only one person left for over 500,000 people [sic]” (Section 303, rural GP group)*

Additionally, the lack of young recruits in primary care was discussed in rural regions and environs. There are many prejudices against being a GP in the country: e.g. little free time, low income, 24/7-avaliability or heavy workloads. GPs were concerned that not enough medical students choose family medicine as their discipline.

### Category 2: Problems due to patient behaviour

We found a clear difference between urban and rural areas regarding the patients’ own assessment of the necessity of their treatment. Patients from rural areas and environs were often reported to consult their GPs too late. Long distances, the reputation in the village community or old age were reported as reasons for missing check-ups. On the other hand, patients in urban areas were considered to consult their GP about banalities e.g. slight colds and sore throats.

*“I often have chest pain, for example, […], but then don’t go to the doctor’s because I say to myself: “Nah, then I have to queue up there.” […] I live far away too, and to go to the doctor’s just for that […], I’d rather not. One says to oneself, okay, it will go away again. […]” (Section 238, rural patient group)*

Patients and GPs from urban areas reported that patients have less confidence in their GP’s abilities. The role of the GP is considered as provider of medical services or supplier of patients for specialists. GPs from the urban areas sometimes felt as though they were not perceived as real doctors.

*“Yes, well, in rural areas a GP is still considered a guide, while in urban areas patients just come and say “I need a referral to a, b, c, d.”, and you won’t see them in your consulting room. (Section 286, urban GP group)*

Due to the mainly urban patients’ low confidence in GPs’ abilities, they often visit specialists single-handedly. This doctor-shopping can lead to the patient being overburdened with the challenge of coordinating his/her own medical care and results in incorrect or unnecessary treatment.

*“Of course [one has] those kind of patients, […] which always go directly to a specialist, where we do not write a referral and therefore do not receive a medical report, then, […] in a worst case scenario, one receives a completely botched patient, who went to the wrong specialist with incorrect symptoms and […] which are overwhelmed with management of their […] diseases” (Section 338, urban GP group)*

### Category 3: Problems through structural inequalities

Patients and GPs from rural areas and environs reported that the low number of GP practices lead to long waiting times and crowded practices. In some districts GP practices do not accept new patients, only in case of death or relocation of regular patients.

*“In our area […] one barely has a chance being accepted as a regular patient of a general practitioner, in every practice you hear “Sorry, we cannot accept any more patients.“. That’s what we hear in our town. There are far too many physicians missing, […]” (Section 249, environs patient group)*

The low number of GPs in the rural areas causes long working hours which burdens the GPs’ work-life balance. Some patients bother their GPs outside the consultation times. GPs from rural areas have to develop strategies to create boundaries between their private life and their work.

*“[…] one has to create clear boundaries […], so that they don’t come Friday night at 10 p.m. and say: “I have had a runny nose for three weeks.” One has to clearly state this. It cannot work that way and they seem to register that relatively quickly.” (Section 309, rural GP group)*

In contrast, the urban areas are dominated by an oversupply of primary care practices. GPs reported high competition and poaching of patients, for example in case of temporary replacement. Furthermore, the high density of primary care practices in urban areas leads to patients changing their GPs more frequently than in rural areas because of minor reasons e.g. other GPs practices having more advanced equipment or not being pleased with the practice assistants. According to the GPs, some patients call on an additional primary care practice nearby their work, giving way to an increased risk of polymedication and interface problems such as deficient knowledge of their health status (concerning e.g. chronic diseases, allergies).

*“[…] but spontaneously I can think of five [physicians] right now, who all say “I cannot go on vacation anymore because I lose 50 patients every time, that I simply cannot afford to lose.”, that definitely happens in [city in northern Germany].” (Section 398, urban GP group)*

Long commutes for GPs and patients are another problem mentioned by GPs and patients. This problem concerned all areas. However, the reasons were different: a lack of parking spots in urban areas versus long distances between GPs’ practices and patients’ homes and poor public transport in rural areas and environs.

*“There is no bus anymore, the small train companies died out long ago.” (Section 356–359, rural GP group)*

*“I [worked] in a rural region […]. I parked in the yard, went in and left again. Here you have to look for a parking spot, park the car, do some little things and have to consider the traffic and whatever else…” (Section 268, urban GP group)*

### Category 4: Problems through the ambulatory reimbursement system

Participants from all areas discussed that the GPs’ budgets do not seem to be adequate. The budget for statutory insured patients available to practices is quickly exhausted by expensive specialists’ medications or many multimorbid patients needing a high amount of treatment (e.g. physical therapy, remedies and aids). As a result, GPs cannot prescribe some medical treatments, loose patients or have to refer their patients to specialists. GPs and patients were very concerned about this situation.

*“[…] he says: “Honestly, I would love to prescribe you [massages], but I have far too many old patients who need them and that I should exercise.”. That’s it. Then I didn’t get massages prescribed from him. I went somewhere else and had massages prescribed.” (Section 96, urban patient group)*

*“[…] sometimes I write referrals, simply to ease the strain on my budget because the specialists have such expensive medications that I, being a general practitioner, cannot afford to prescribe them anymore. It’s that simple. I often write referrals […] to neurologists for this reason, it isn’t possible any other way.” (Section 70, rural GP group)*

GPs from all areas stated that some consultations were not appropriately reimbursed. These included, for example, conversations with patients about relationship problems, work-related problems, legal issues, child raising, organizing care or applying for rehabilitation. Diagnosis and treatment of patients with mental disorders were also discussed explicitly.

*“Yes, sometimes it’s simply life-coaching, […] people… ask whether they should sell their house after a divorce, or if they should quit their job or not quit their job, or they face life-changing decisions which they seem to not be able to make on their own. I find it astonishing.” (Section 161–163, urban GP group)*

*“[…] Well, starting at severe depression and psychosis, one does get an appointment for the patients, but not for all our burn-out patients, where one would really like to have a specialist’s opinion on whether or not it is a depression or just shirking, etc. So, sometimes one is dependent on specialist colleagues. We are left alone. We also feel misused and, sometimes, I have the feeling that once we have convinced the patients that they should see a psychiatrist, half the work has already been done. […]” (Section382, rural GP group)*

### Category 5: Problems with specialist care

Participants reported a lack of specialists in all regions leading to long waits for appointments, which could be several months in urban areas or up to over a year in rural areas. Especially waiting times for appointments with psychiatrists and psychotherapists were reported to be very long. GPs have different strategies to forgo this problem. Some GPs contact specialists directly via telephone to make appointments for their patients. In some cases, they declared their patients as an emergency or send them to hospitals so that the patients are examined earlier. These strategies were described as helpful, but time consuming. Some patients reported that they bridge long waiting times by self-medicating, which might not be the most favorable strategy. They also complain about long waiting times for appointments when pain or other complaints are not at the highest level or acute.

*“And, or one uses the telephone and sometimes has to declare the patients more ill than they really are so that they get an appointment quickly, otherwise it doesn’t work out at all… Even then… one cannot constantly call someone and declare catastrophes […] (Section 238, environ GP group)*

*“The [orthopedists] are completely booked, it takes three to four weeks […] of course it’s annoying, […], running around in pain the entire time, so you take random pain medication, which in turn causes stomach problems and when you go to your appointment after 4 weeks, then the pain might not even be so bad anymore […].” (Section 280, urban patient group)*

Furthermore, GPs had to assume specialists’ duties. They described this problem as a great challenge which is interesting and overwhelming at the same time. The GPs were afraid that they could not treat their patients adequately. Nevertheless, they assume these duties (e.g. minor surgical interventions, children checkups or psychosomatic/psychological care).

*“[…] And in other cases, we have to bridge the waiting times until the necessary therapy in whatever form (to solidify an inpatient treatment or as outpatient therapy) can be administered through specialists. (Section 382–384, rural GP group)*

Additionally, communication problems between specialists and GPs were reported. Specialists do not inform or inadequately inform the patients’ GPs. Participants from urban areas and environs reported that some specialists never send medical reports or that the letters are incomplete (e.g. missing medical diagnoses). In this context, patients from urban areas or environs mentioned that they visit some specialists without a referral from their GP which could increase the problem.

### Category 6: Problems with inpatient care

GPs reported that they had to assume the consequences of problems with inpatient care. GPs from all areas stated that they had to provide follow-up care for patients discharged with still healing wounds.

*“Discharge management is a huge problem, you know? Patients often receive a “bloody discharge” on Friday afternoons… with luck medications are provided until Sunday evening. There is no way to access the medical report, the patient is at home, the daughter is standing in the practice […] at 1 p.m. on Friday.” (Section 442, environ GP group)*

One problem was mentioned only by participants from rural areas. Staff shortage in hospitals reduces the quality of inpatient care. GPs and patients presumed that the hospitals had problems with hiring staff. Many physician positions in the hospitals were occupied by physicians from abroad and with temporary contracts.

*“[…] the [hospital] in [rural district in Lower Saxony]. I know that they have extreme problems filling the vacant positions because I have talked to one of the medical directors on the phone, who said that no one wants to live in the country, which is why so many physicians from abroad are hired, then there are language barriers, and they have to be integrated […] (Section 407, rural GP group)*

GPs from all areas complained about the lack of cooperation and communication between GPs and hospitals. For questions about medical diagnostics and therapies, it is difficult to find a contact person in the hospital. Furthermore, there is generally no information exchange between hospitals and GPs. This results in such things as unnecessary repetitions of diagnostics.

*“One could certainly make things easier, if the hospital-GP cooperation was improved. It is always a bit annoying, always having to call, but somehow I always had the feeling that I was properly announcing the patient. And now it’s pfft, send him/her over and pfft. Then the colleague there starts at the very beginning again, has no information whatsoever and I don’t like that.” (Section 322, rural GP group)*

Also, the discharge reports from hospitals were partially described as incorrect or missing. The reports consisted partly of nonsensical text blocks, and sometimes information about the anamneses or the medication was missing. Moreover the discharge reports were delivered too late. GPs have to treat/care for the patients despite lacking information, which is, in many cases, made unnecessarily difficult for them.

Another problem mentioned is the level/number of prescribed medications from the hospital. GPs from environs and urban areas reported that the prescribed medications from the hospitals are often very expensive or the number of medications increased after hospitalizations. The GPs had to reduce the medication to an outpatient level, which often involves extensive discussion with the patients.

*“Something that is very time consuming is the transition from inpatient to outpatient care. To get the patient back to the point where he is treatable as an outpatient and doesn’t have to live with just the hospital’s ideas. […] to get rid of the hundreds of medications. One has to bring the patient to an outpatient level.” (Section 86–88, environ GP group)*

## Discussion

### Main findings

Our results comprehensively mirror both the providers’ (GPs) and consumers’ (patients) perspective on problems in ambulatory care and their regional variations in northern Germany. We found differences and commonalities between rural and urban regions. While some of the identified problem areas (e. g. aging society, lack of young recruits) are already well proven others (e. g. patients’ judgment on treatment necessity, poaching of patients) lacked empirical data until now. The main problems mentioned in rural areas were the insufficient physician density and lack of young recruits in primary care practices resulting in an increased workload for existing GPs. The urban areas were affected more by a high physician density associated with high competition between GPs and a high fluctuation of patients, as well as problems with the perceived low status of GPs. Problems in all regions concerned the ambulatory reimbursement system, especially inadequate GPs’ budgets and insufficiently compensated consultations, the specialist care (particularly the lack of specialists) and the inpatient care. Most problems were mentioned by GPs and patients alike, but some, e.g. high competition rates in urban regions, inadequate compensation of consultations, lacking flow of information between specialists and GPs and problems with inpatient care, were only mentioned by GPs.

### Strengths and limitations

To our knowledge, this is the first qualitative study analyzing regional variations of perceived problems in ambulatory care from the perspective of GPs and their patients in northern Germany. We took into account both the providers’ and the consumers’ perspectives, a strategy which helps to explore complementary or even contradictory views on certain problems [34]. The problem areas identified in our study might vary from results of studies in other countries, due to different health care systems. Nevertheless, other countries reported similar problems [9–14].

Our recruitment method allowed the self-selection of participants which might have biased our findings towards a more negative or positive direction. General practitioners and patients with a more positive or negative attitude towards their medical practice or their region of practice could have been more motivated to take part in our study. One cannot rule out that the proportion of heavy users of the healthcare system or patients wanting to do their GP a favor could be elevated in our focus groups. Also GPs and patients, who were particularly affected by problems in ambulatory care, could have been more motivated to participate. As all GPs and patients stated positive and negative views about primary care and the health care system in general, we do not consider our data to be biased into an overly positive or negative direction and believe it represents a variety of experiences. To reduce the risk of bias even further, we took care to include male and female GPs, with longer and shorter durations of practice experience and male and female patients representing the whole age spectrum.

Our focus group participants show differences especially in marital status and employment status with people from rural regions being more often married and less often employed than participants from environs or urban areas. Findings on the influence of marital and employment status on self-rated health and health care seeking behavior (in Germany) do not seem to be conclusive (e.g. [35–37]. Given that, we feel that we cannot make any assumptions about the possible influence of the participant characteristics on our results. For every region there was considerable variation in patient characteristics concerning marital status, education, employment status, age, gender, chronic diseases and degree of disability with almost no characteristic not being represented. Either way, as the aim of our sampling was to maximize the variation of accounts of our focus group participants and not to achieve representativeness in the queried group we do not feel the slight differences in characteristics proportions to be skewing our results.

We have not been able to recruit many oldest-old patients (aged 80+, *N* = 2) in our study. This might be due to our recruiting approach (asking GPs to recruit patients visiting their practices, excluding nursing home residents) and the need for participants to be mobile enough to get to the location where the focus groups took place. Including more oldest-old patients (and nursing home residents) tending to be the greatest users of GPs, specialist services and inpatient care might have resulted in even more pronounced descriptions of perceived problems in ambulatory care. To shed more light on problems in ambulatory care perceived by these special groups of people further studies using different ways of approaching potential informants (for example focus groups in nursing homes, face-to-face interviews at the private homes of the oldest-old persons) are needed.

### Discussion of results and comparison with existing literature

GPs and patients from rural areas intensely discussed the lack of young recruits in primary care in the focus groups. The impending lack of primary care supply in rural areas was associated with long waiting times and crowded practices. Especially the GPs feared an increasing workload which they would no longer be able to cope with. Kopetsch [4] predicted a total of 23,768 retiring GPs in Germany until 2020. The expected number of new primary care practices will be not enough to fill this gap [4, 38]. In this context, it will be very important to strengthen the job satisfaction of GPs and to improve the image of primary care, especially in rural areas. Further studies reported GP dissatisfaction regarding workload, income, administrative tasks and bureaucracy [39, 40]. Löffler et al. identified factors that increase the job satisfaction of GPs in Mecklenburg-Western Pomerania: a good doctor-patient relationship, fair pay and the variety of reasons for doctor-patient consultations [41]. Pohontsch and colleagues found rural GPs to be satisfied with their working conditions due to similar reasons (Pohontsch et al.; unpublished manuscript, under review).

GPs from rural areas reported taking over duties and responsibilities of the missing specialists in their areas, other studies show similar results. Steinhäuser et al. [23] found that GPs in rural regions perform more and different procedures than GPs from urban areas, a finding that is also true for other countries [42]. Voices have been raised requesting changes in the federal requirement planning concerning specialists for somatic and mental illnesses [43], which would help to relieve especially rural GPs from the burden to step in for “missing” specialists.

These aspects should be considered when planning initiatives or marketing activities promoting the field of (rural) primary care for young medical recruits and decreasing prejudices against working in rural areas. National and international university initiatives, as well as programs for the recruitment of primary care physicians already exist and have proven to be useful [44], but there is potential to extend and improve them [45]. In Germany initiatives exist to (financially) support students choosing to absolve internships in general practice and reoccupation of free licenses for registered physicians in rural regions by GPs and specialists (for example [43]). Delegation models for non-physician healthcare staff were discussed and implemented [46], these models could unburden the GPs especially in rural areas with large distances between practices. An increased density of general practitioners could solve the problem of long distances for both sides, the general practitioner would profit in case of house calls and patients could access health care more easily, thus perhaps reducing the number of house calls. The “DB medibus” initiative providing former public transport busses refitted to function as fully equipped practices visiting sparsely populated and undersupplied regions and small busses transporting patients with restricted mobility (without other transport options like public transportation or family or friends providing transport) can be considered as a supporting measure and operates in a pilot phase since 2016 [47]. Other countries already profit from the implementation of telemedicine in rural areas [48], while telemedicine is not integrated into nationwide regular health services in Germany yet [49]. There are some projects studying telemedicine in general practice, often telemedic monitoring [48]. Further studies and efforts are needed to evaluate existing concepts and promote the use of telemedic concepts in undersupplied areas which is up to now somewhat restricted by legal, technical and trust challenges [49].

GPs reported different consultation behaviors of their patients depending on the region of registration, with urban patients consulting their GPs for banalities while rural patients seem to delay visits to the general practice. Studies from other countries, e.g. Sweden, Australia and Scotland, find similar results, reporting patients in rural regions to consult primary health care less frequently than urban patients for all kinds of conditions [50–54], with urban patients being more demanding and showing a more ‘consumerist’ orientation [50]. This could be partly explained by the fact that a high supply usually leads to higher utilization rates. A higher density of physicians leads to increased utilization of their services [7] and a rather slight reduction of ambulatory care sensitive hospitalizations [18]. Grobe et al. shows a higher number of billed treatment cases and higher treatment costs for patients from city states (e. g. Hamburg, Bremen) [55]. The reported higher fluctuation rates of patients and competition for patients between GPs in urban regions might interrelate with each other. This could be due to the fact that there is a high GP density in urban areas and patients seem to trust their GPs less than in urban regions. The reported decreased trust in GPs’ abilities might also further patients’ tendency to change GPs, if not satisfied. Although the GPs’ subjective reports state that patients tend to change GPs often in urban regions, statistics show that doctor-shopping is not patients’ usual behavior [55–57]. The abovementioned problems faced by urban GPs (patient fluctuation, poaching of patients) could be handled by strengthening the role of the GP as a coordinator in the German health care system. GP-centred health care contracts [“Hausarztzentrierte Versorgung” (HZV), general practitioner-centered integrated care] as they were offered by the German statutory health care funds could probably represent a solution. The evaluation of these contracts by Klingenberg et al. found that 70.0% of the survey’s participants (GPs) evaluated the HZV positive in general, 60.1% felt it strengthens their role as a GP. Positive effects were also seen concerning coordination of care and cooperation with patients [58]. Laux et al. reported in their extensive evaluation of the GP-centered health care in southern Germany fewer contacts with the GP and fewer referrals to specialists in the group with GP-centered health care [59]. The challenge to reduce potentially harmful delays of health care seeking behavior in rural patients, which is pronounced by worse accessibility of rural healthcare, remains [50].

The existence of interface problems between GPs, specialists and hospitals is a well-known fact, e.g. [60, 61]. Electronic health cards as means to record prescriptions, results of diagnostic measures and medication plans could be helpful. Study results indicate a high acceptance of an emergency data set (information on prior diagnoses, medications, allergies, medical implants and other emergency related information) by primary care physicians, clinicians, emergency physicians and paramedics [62]. This could be expanded to include other data as well. However this approach still gives rise to concerns about data safety and confidentiality. Until now, the responsibility for the validity and up-to-dateness of the data is not clarified. Interface problems and paucity of specialists could also be solved by repealing the strict separation between ambulatory and stationary sectors. This could facilitate the use of specialized medical structures (eg. in hospital or outpatient institutions) and, thus, compensate for the lack of specialists, especially in rural areas [63].

Blum and Löffert showed that hospitals in rural regions have the biggest share of vacant physician-positions in Germany. Paucity of physicians leads to problems in organizing work shifts, overworks physicians and impairs patient care. 5% of all positions in rural hospitals stay vacant [49]. One can assume that, from the potential applicants’ point of view, lack of infrastructure (e.g. child care facilities, cultural life and shopping facilities) and career prospects, significantly reduce the attractiveness of working in rural hospitals [48]. Together with our findings on interface and patient care problems reported by GPs and patients, this indicates a need to increase the attractiveness of working in rural hospitals e.g. by increasing financial incentives or incorporating family-friendly policies.

## Conclusion

Problems in ambulatory care, from the perspective of GPs and their patients in our study, showed some regional variations. While many problems arise in urban regions as well as in rural regions, our results support the notion that there is an urgent need for action in rural areas. Possible measures include the support of telemedicine, mobile practices, and delegation of medical services and reoccupation of vacant practices. The attractiveness of working in rural areas for general practitioners, specialists and clinicians must be increased by consolidating and expanding rural infrastructure (e.g. child care and cultural life) and changes in the federal requirements planning might be needed. All regions are concerned with the ambulatory reimbursement system, especially GPs’ inadequate budgets and insufficiently reimbursed consultations, as well as problems with specialist care and inpatient care. The ambulatory compensation system should be examined regarding these reported inequalities, especially the different reasons for consultations. There is an overall demand to enhance the cooperation between GPs, specialists and inpatient care, for example through facilitating communication via electronic health cards.

Further studies are needed to quantify the extent of the identified problems and differences. The second part of our study, a survey to quantify regional differences in primary health care in northern Germany, is currently under way [29]. Independent of the survey’s results, the presented results indicate the need to balance these variations between the regions. For this, different actors are required: politics, self-management of ambulatory care, concerned individuals (doctor and patient) and, ultimately, those responsible for medical offspring. Existing measures and interventions (as already described above) need to be further developed and evaluated.

## Abbreviations

GP: General practitioner
HZV: Hausarztzentrierte Versorgung (general practitioner-centered integrated care)
